# Improved remote sensing ecological index for monitoring urban sustainability: 100 resilient cities vs non-resilient cities in South Asia

**DOI:** 10.1007/s10661-026-15156-w

**Published:** 2026-03-13

**Authors:** Jayanta Biswas, Niloy Biswas

**Affiliations:** 1 Department of Earth Sciences, University of Memphis, Memphis, TN USA 38152; 2 Urban and Rural Planning Discipline, Khulna University, Khulna, 9208 Bangladesh

**Keywords:** Remote sensing ecological index (RSEI), Urban resilience, Urban sustainability, 100 resilient cities, South Asia

## Abstract

This study assesses ecological change in two 100 resilient cities (100RC) (Pune, Surat) and two non-100RC cities (Chattogram, Nagpur) in South Asia using an improved remote sensing ecological index (RSEI). The enhanced model incorporates six indicators composite vegetation index (CVI), wetness, normalized difference build-up and bare soil index (NDBSI), impervious surface index (ISI), urban index (UI), and land surface temperature (LST)—with entropy-based weighting and a moving window approach to overcome traditional PCA limitations. The results reveal apparent differences between 100RC and non-100RC cities. Non-100RC cities exhibited high volatility, characterized by sharp ecological declines, partial recoveries, and subsequent collapses. In contrast, 100RC cities followed steadier but persistent downward trajectories: Surat exhibited a monotonic decline, while Pune improved until 2022 before declining in 2024. Hotspot analysis revealed that cold spots expanded dynamically in non-100RC cities, whereas they spread gradually in 100RC cities. Moran’s *I* confirmed strong spatial clustering (0.89–0.97) across all cities. A two-way ANOVA revealed significant group, year, and interaction effects (*p* < 0.001). These findings suggest that resilience frameworks reduce ecological instability but are insufficient to halt structural degradation. Strengthening urban resilience requires more rigorous policy enforcement, continuous monitoring, and ecological restoration to achieve sustainable ecological outcomes.

##  Introduction

For human existence, the ecological environment serves as a fundamental foundation. It is directly linked to human health and well-being, socioeconomic development, and sustainable growth (G. Zhang & Kuang, 2025). Rapid urban and industrial development has had a detrimental impact on the ecological spaces in urban areas. Therefore, accurately assessing the ecological environment has become a critical issue (Chen et al., 2023; McGranahan & Satterthwaite, 2014; Wang et al., 2023). Accurately assessing ecological conditions has therefore become a prerequisite for evidence-based planning and monitoring urban sustainability.

The government, international organizations, and non-governmental organizations (NGOs) have launched several initiatives aimed at creating sustainable and resilient cities. One such initiative is 100 resilient cities (100RC), pioneered by The Rockefeller Foundation in 2013. Its goals are to promote a holistic resilience framework designed to strengthen cities against both acute shocks (such as floods and earthquakes) and chronic stresses (such as pollution, infrastructure strain, and social inequality) s (Allan & Bryant, 2011). While these frameworks have advanced the governance and infrastructural dimensions of resilience, their environmental outcomes remain insufficiently quantified. The ecological dimension of resilience, encompassing ecosystem health, green infrastructure, and environmental quality, is often treated conceptually rather than empirically measured.

Urban resilience and environmental sustainability are inherently interconnected aspects of sustainable urban development. A crucial yet often overlooked pillar of this resilience is the urban environment, encompassing ecological health, green infrastructure, and environmental quality. Research indicates that resilient cities often incorporate nature-based solutions and adaptive environmental governance to mitigate the effects of climate extremes, reduce urban heat, and manage flood risks (Meerow et al., 2016; Sharifi, 2016). For example, expanding urban green space and preserving ecological corridors can enhance stormwater absorption, mitigate the urban heat island effect, and improve overall public health, thereby strengthening a city’s long-term adaptive capacity (Elmqvist et al., 2019). This disconnect highlights a crucial research gap: how can resilience strategies be evaluated using objective, spatially consistent ecological indicators? Bridging this gap requires tools that can capture multi-dimensional environmental dynamics using accessible data.

Traditionally, one of the most commonly used models for evaluating ecological conditions is the Pressure-State-Response (PSR) model developed by the Organization for Economic Cooperation and Development (OECD) and the United Nations Environment Program (UNEP) (Tavosi et al., 2025; G. Zhang & Kuang, 2025). The PSR models include multiple dimensions, such as pressure, state, and response. However, gathering these variables often faces challenges like data scarcity, difficulty in obtaining data, and high costs.

To address data acquisition, scarcity, complexity, and spatial limitations, Xu (2013) developed the Remote sensing ecological index (RSEI). The RSEI model incorporates four ecological indicators: greenness, wetness, dryness, and heat. The RSEI index effectively addresses the challenges of data collection and the construction of complex indicators, enabling broader application. However, the PCA-based weighting used in conventional RSEI can vary across regions and years, often ignoring water bodies and under-representing heterogeneous urban surfaces (Biswas, 2025; Wang et al., 2023; Zhu et al., 2020). Building on recent advances in entropy weighting and moving-window analysis (Biswas, 2025; Liao & Jiang, 2020; Wang et al., 2023; G. Zhang & Kuang, 2025), this study develops an improved RSEI that expands the set of ecological indicators and enhances spatial adaptability. Recent syntheses argue that resilience frameworks improve adaptive capacity but often lack quantitative environmental performance metrics to verify outcomes on the ground (Elmqvist et al., 2019; Meerow et al., 2016; Sharifi, 2016). Remote-sensing indices such as the RSEI can bridge this gap by translating ecosystem condition into spatially explicit measures that are trackable over time. Building on this perspective, we use an improved RSEI to empirically test how resilience initiatives relate to ecological stability in fast-urbanizing South Asian cities.

Resilience planning not only addresses challenges related to infrastructure and governance but also creates opportunities to safeguard ecological systems, thereby ensuring long-term urban health and sustainability. By linking resilience frameworks with environmental performance metrics, such as the RSEI, we can adopt a comprehensive approach to evaluate whether resilience planning efforts are yielding tangible ecological outcomes.

This study aims to evaluate ecological sustainability in two 100RC cities (Pune, Surat) and two non-100RC cities (Chattogram, Nagpur) across 2016–2024 using this improved RSEI, and to determine how resilience frameworks influence ecological stability in rapidly urbanizing South Asia. While the 100RC initiative provides a conceptual and policy foundation for urban resilience, it lacks operational tools for environmental monitoring and assessment. The RSEI offers a complementary, data-driven perspective to assess and compare the environmental dimension of resilience between 100RC cities (Pune and Surat) and non-100RC cities (Nagpur and Chattogram). This integrated approach highlights spatial disparities in ecological health that may be overlooked by policy-driven frameworks alone (Meerow et al., 2016; Sharifi, 2016; H. Xu et al., 2019).

## Study area

**Fig. 1:**
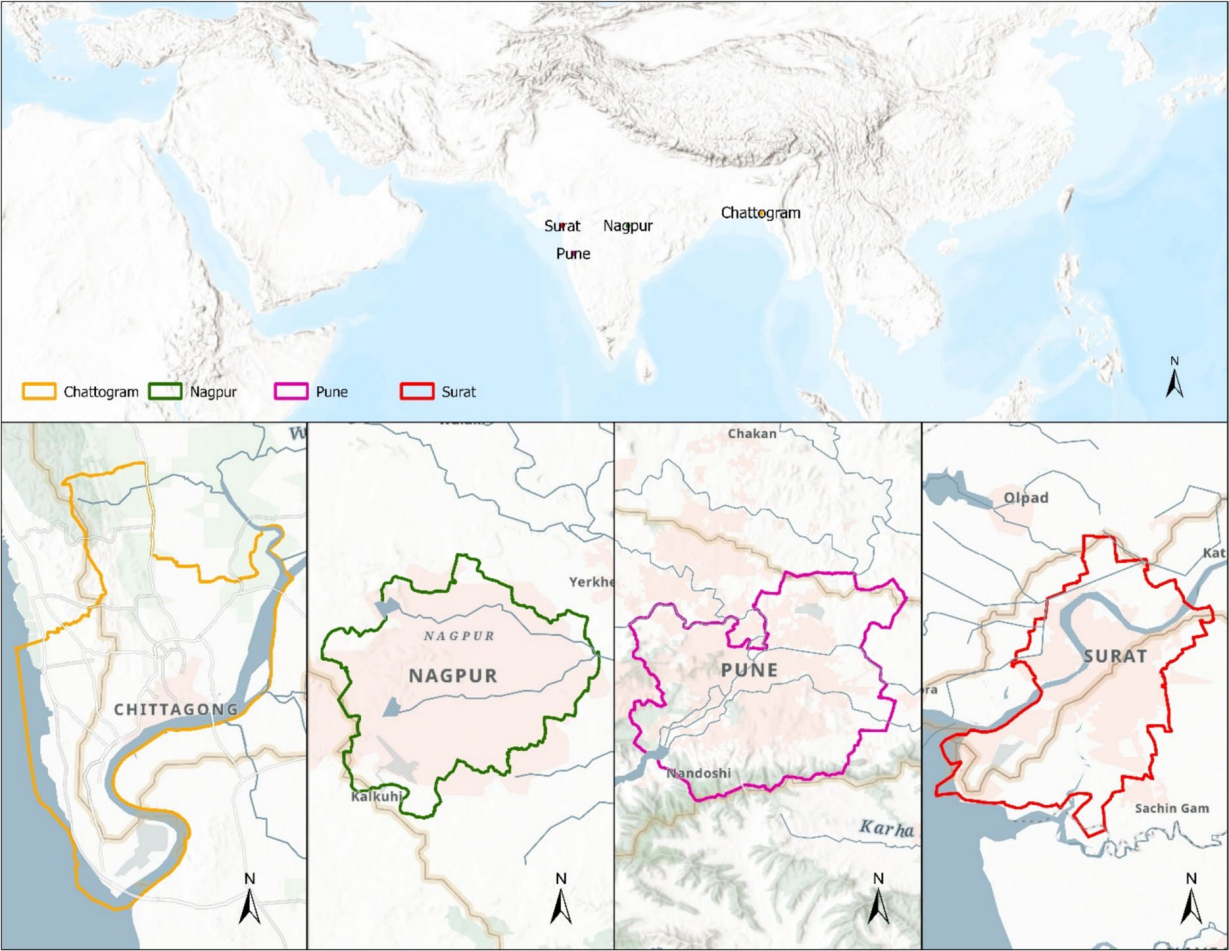
Study area maps

This study examines four rapidly urbanizing cities in South Asia: Chattogram (Bangladesh), Nagpur, Pune, and Surat (India) (see Fig. 1). These cities were selected to offer a comparative perspective on urban ecological conditions across various resilience planning contexts. Of these, Surat and Pune are part of the 100RC network, while Chattogram and Nagpur are not. All four cities are undergoing significant urban expansion, infrastructure development, and facing environmental challenges, making them pertinent cases for evaluating ecological health through remote sensing methods. Table 1 provides a quick description of the selected cities.

**Table 1:** Quick description

City	Country	Coastal	Elevation (m)	Climate	100RC member
Chattogram	Bangladesh	Yes	~ 12	Tropical monsoon	No
Surat	India	Yes	~ 13	Tropical savanna	Yes
Pune	India	No	~ 560	Tropical wet/dry	Yes
Nagpur	India	No	~ 310	Tropical wet/dry	No

Chattogram, situated on the southeastern coast of Bangladesh, is the country’s second-largest city and its principal seaport. The city has encroached upon natural drainage systems and low-lying areas, leading to increased environmental degradation and ecological stress. Furthermore, the rapid, uncoordinated urban and industrial growth in Chattogram is placing significant strain on the ecological environment. Chattogram’s development efforts also fall short of aligning with principles of environmental sustainability, which may lead to adverse effects on the environment, human health, biodiversity, and climate (Kaiser, 2023).

Surat, located on the west coast of India in the state of Gujarat, is a prominent industrial hub known for its textile and diamond industries. Despite facing challenges such as flood risks and rapid land conversion, Surat has garnered international acclaim for its resilience initiatives and is among the select Indian cities participating in the 100RC initiative. The city’s first resilience strategy was published in April 2017.

Pune, situated in the western Indian state of Maharashtra, is a highland city renowned for its educational institutions, IT sector, and automotive industries. Pune is particularly vulnerable to severe disturbances, such as heavy rainfall, flooding, and disease outbreaks within the urban core. Additionally, it faces chronic stresses like water pollution, challenges in managing wastewater and solid waste, a shortage of affordable housing, and transportation difficulties. Pune’s Resilience Strategy is centered around three critical areas for enhancement, taking into account Pune’s current development trends and projected growth: urban mobility, urban environment (which includes water body management and biodiversity conservation), and the urban economy, with a particular emphasis on the informal sector.

Nagpur, situated in central India, presents a contrasting scenario. Although it is not part of the 100RC initiative, the city is recognized as a leader in sustainable development amid India’s rapid urbanization. Selected under the government’s “smart city” project, Nagpur confronts numerous urban challenges that the current development plan fails to address adequately. The haphazard and rapid infrastructural development, which lacks a comprehensive strategy, is leading to urban sprawl that undermines the city’s green infrastructure (Dhyani et al., 2018). This approach overlooks the benefits of nature and ecosystem services that could alleviate and mitigate urban stress.

Together, these four cities form two paired comparisons—coastal Chattogram vs. coastal Surat, and inland Nagpur vs. inland Pune—allowing us to “compare apples with apples” in terms of climate and geography while isolating differences related to participation in the 100RC network and resilience planning. In summary, the four cities offer complementary contrasts—coastal vs. inland and resilient vs. non-resilient—creating an ideal setting to explore how governance frameworks influence ecological change.

## Methodology

### Data and data processing

This study used surface reflectance (SR) and top of atmospheric (TOA) product of Landsat 8 OLI imagery from 2016 to 2024 in a 2-year gap to identify the ecological environment change in 100RC and non-100RC cities. This study utilized the Google Earth Engine (GEE) platform to calculate the indices required for RSEI calculation. The process of data collection and processing is explained below:

1. Selection of study period and data: Landsat-8 OLI and TOA images for 2016, 2018, 2020, 2022, and 2024 were collected for the study period using the Google Earth Engine Python package (geemap).
2. Cloud masking: Images with less than 5% cloud cover were retained to ensure the accuracy of vegetation and urban surface measurement.
3. Radiometric calibration: The surface reflectance data were calibrated using the formula 0.0000275 × DN + offset to produce the SR product, balancing precision with practicality.
4. Composite stacking: For each year, cloud-free images were stacked using the median function to reduce noise and ensure consistency in temporal analysis.
5. AOI clipping: The stacked images were clipped to the AOI for each city to focus on the urban area of interest.

### Calculation of ecological indices in GEE

**Fig. 2:**
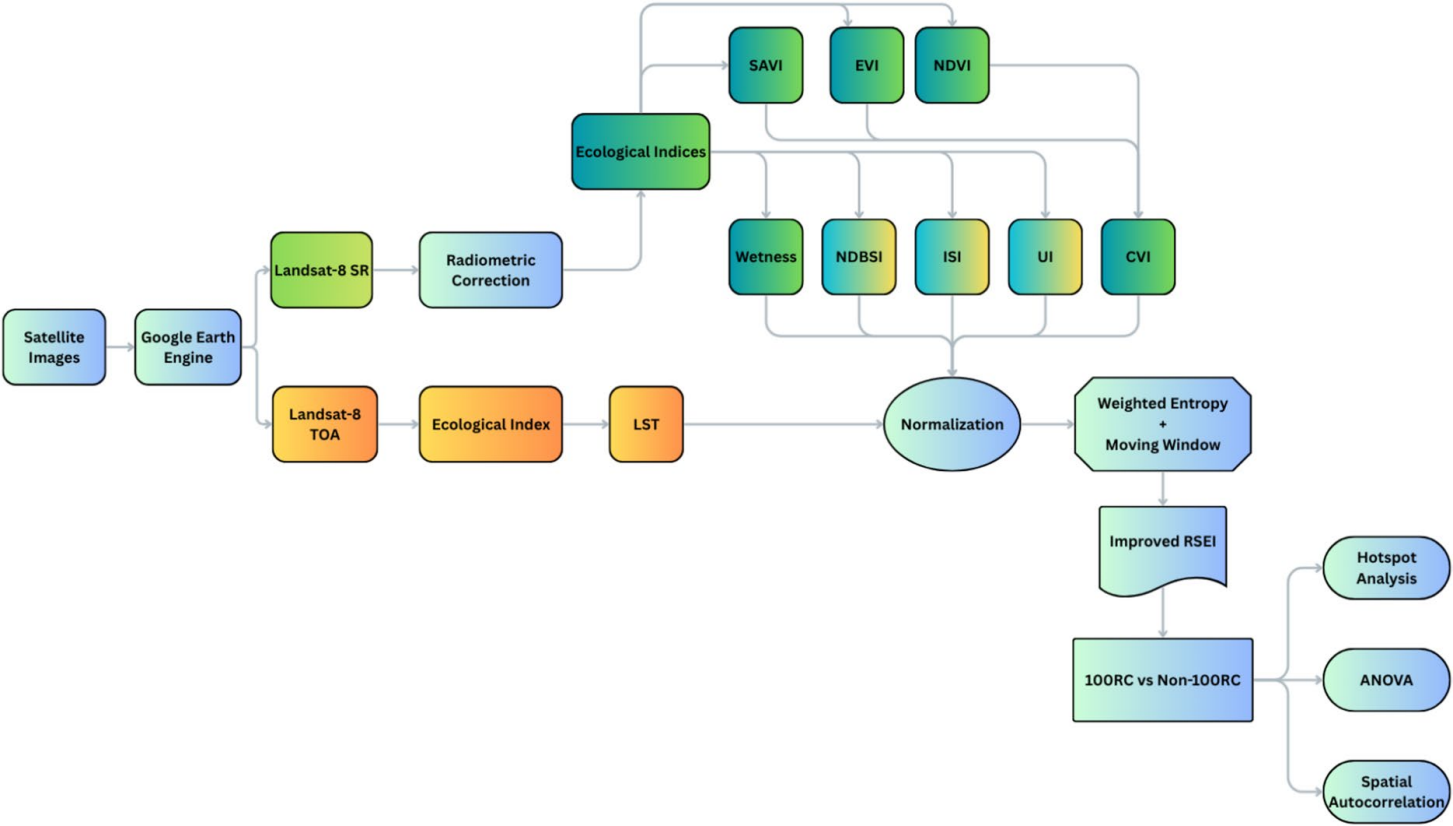
Methodological framework

The Landsat 8 SR product was used to calculate all the indices except land surface temperature (LST). LST was computed using the TOA product, as this product provides the raw thermal radiance values needed to begin the LST retrieval process. Besides that, TOA products are globally consistent and atmospherically corrected to a degree, making them reliable for comparing across time and space. Table 2 provides the equation and an overview of the indices used in this study, and Fig. 2 illustrates the methodological flow.

**Table 2:** Spectral indices equations

Index	Data	Equation	Citation
NDVI	Landsat-8 SR	(NIR − Red)⁄(NIR + Red)	(Rouse, 1973)
EVI		2.5 × ((NIR − Red)⁄(NIR + 6 × Red − 7.5 × Blue + 1))	(A. Huete et al., 2002)
SAVI		((NIR − Red)⁄(NIR + Red + 0.5)) × 1.5	(A. R. Huete, 1988)
CVI		NDVI × EVI × SAVI	Author defined
Wetness		0.1509 × B2 + 0.1973 × B3 + 0.3279 × B4 + 0.3406 × B5 − 0.7112 × B6 − 0.4572 × B7	(Crist & Cicone, 1984)
NDBSI		(BI + SI)⁄2, where BI = (SWIR − NIR)/(SWIR + NIR), SI = (SWIR − SWIR2)/(SWIR + SWIR2)	(Zha et al., 2003)
NDBI		(SWIR − NIR)⁄(SWIR + NIR)	(Zha et al., 2003)
ISI		(NDBI − NDVI)⁄(NDBI + NDVI)	(H. Xu, 2007)
UI		(SWIR − NIR)⁄(SWIR + NIR)	(Weng et al., 2004)
LST	Landsat-8 TOA	(TB/(1 + (λ × TB/ρ) × ln(ε))) − 273.15 (from Band 10)	(Sobrino et al., 2004) [Parameters updated using USGS Landsat 8 product]

*NDVI* normalized difference vegetation index, *EVI* enhanced vegetation index, *SAVI* soil adjusted vegetation index, *CVI* composite vegetation index, *NDBSI* normalized difference build-up and bare soil index, *NDBI* normalized difference build-up index, *ISI* impervious surface index, *UI* urban index, *LST* land surface temperature, *NIR *SR_B5, Red = SR_B4, Blue = SR_B2, SWIR = SR_B6, SWIR2 = SR_B7; λ = 10.8 µm,* ρ* = 1.438 × 10⁻^2^ mK in LST equation

## Traditional and improved RSEI model

### Traditional model

Traditionally, the RSEI model uses four environmental variables: greenness (NDVI), wetness, dryness (NDBSI), and heat (LST) from remote sensing data for calculation. The weight of each variable on the environment was measured using principal component analysis (PCA) (Xu, 2013). Initially, the first principal component is calculated to estimate the initial RSEI value, and then it is normalized using the minimum and maximum RSEI. The reported RSEI value ranges from 0 to 1, where a larger value indicates better environmental quality. The impact of each component on the ecological components was expressed by the calculation of PCA (H. Q. Xu, 2013) as displayed in Eq. 1. RSEI can be calculated using Eqs. 2 and 3, where PC1 is the first principal component calculated from the PCA.

1 $$RSEI=f\left(Greeness,\;Wetness,\;Dryness,\;Heat\right)$$

2 $${RSEI}_{0}=1-{}{PC}1\left(\mathrm{NDVI},\text{ Wetness},\text{ LST},\text{ NDBSI}\right)$$

3 $$RSEI= {(RSEI}_{0}-{RSEI}_{0\_\mathrm{min}})/{(RSEI}_{0\_\mathrm{max}}-{RSEI}_{0\_\mathrm{min}})$$

Researchers are measuring RSEI using the PCA method for predicting the impact of population growth and impervious surface on the environment (H. Xu et al., 2018), assessing the ecological vulnerability (Kamran & Yamamoto, 2023), the impact of mining activity on the ecology (Zhu et al., 2020), and many more sectors. However, the PCA method has limitations. The first limitation of this method is that it cannot be applied uniformly (Tang et al., 2015; Wang et al., 2023). Additionally, in applied research, the first principal component typically contributes between 60 and 90%, without a fixed standard, which does not guarantee a high contribution rate or minimal data loss. The major problem is that to avoid the impact of water, it excluded water bodies from the analysis (Chavez, 1996; Zhu et al., 2020). As we know, water is one of the most significant mitigators of heat, and it has a considerable impact on the ecology. Excluding water bodies from the analysis means that the PCA method does not consider all environmental elements during RSEI calculation.

### Modified model

To overcome the limitations of PCA methods, some studies have tried an entropy-based weighted method. The entropy-based RSEI model considers the complete element through a weighted method. Notably, the PCA-based RSEI model fails to account for critical variables such as waterbodies and relies on inconsistent, subjective weighting schemes, which can undermine both accuracy and comparability (Liao & Jiang, 2020; Wang et al., 2023). Recently, Biswas (2025) an entropy-based ecological index was used to monitor urban environmental degradation in 12 Bangladeshi cities. Liao and Jiang (2020) used this method to evaluate spatiotemporal variation in the environmental quality in China, and Wang et al. (2023) tried to further improve the weighted entropy model by incorporating a moving window to simulate the land surface eco-environment.

4 $${E}_{j}=-\mathrm{ln}{\left(n\right)}^{-1}{\sum }_{i=1}^{n}{P}_{ij}\mathit{ln}\left({P}_{ij}\right)$$

The entropy weighted method is used to determine the objective weights of four ecological indicators using Eq. 4. Where E_j_ is the information entropy of each indicator, n is the number of different values in each indicator, and P_ij_ is the probability that the ith value occurs in the jth indicator. Then, the entropy weight of the j-th indicator can be expressed as follows:

5 $${W}_{j}= \frac{1-{E}_{j}}{m-\sum {E}_{j}}\left(j=\mathrm{1,2},\dots m\right)$$

Here, W_j_ is the weight of each indicator, and m is the number of ecological indicators. Before the entropy weighted method is calculated, the ecological indicators were discriminated (with forward normalization for the positive indicators and inverse normalization for the negative indicators) to ensure the reliability of the results using Eqs. 6 and 7.

6 $${Y}_{ij}={X}_{ij}-\mathrm{min}({X}_{ij})/{\mathrm{max}(X}_{ij})-\mathrm{min}({X}_{ij})$$

7 $${Y}_{ij}={\mathrm{max}(X}_{ij})-{X}_{ij}/{\mathrm{max}(X}_{ij})-\mathrm{min}({X}_{ij})$$

NDVI and wetness indicators were normalized using Eq. 6; on the other hand, NDBSI and LST were normalized using Eq. 7. Finally, Eq. 8 for RSEI estimation.

8 $$RSEI= {W}_{1(i,j)}\times NDVI+{W}_{2(i,j)}\times Wetness+{W}_{3(i,j)}\times NDBSI+{W}_{4(i,j)}\times LST$$

Besides the weighted entropy model, Gong et al. (2025) an autoencoder neural network with a long short-term memory (LSTM) deep learning model was used to evaluate the RSEI. Alqadhi et al. (2024) used PCA, fuzzy logic, and fuzzy analytic hierarchy process (FAHP), and combined these three models using fuzzy logic again, and developed an integrated model for obtaining RSEI.

### Improved model

Methodologically, our design follows advances that replace PCA’s fixed loadings with objective entropy weighting and introduce local moving-window computations to capture fine-scale heterogeneity (Liao & Jiang, 2020; Wang et al., 2023; G. Zhang & Kuang, 2025; Zhu et al., 2020). We also align with recent efforts to expand indicator sets beyond greenness–wetness–dryness–heat to better resolve better urban surfaces (ISI, UI) and vegetation synergy (CVI) (Weng, 2009; H. Xu, 2007; T. Zhang et al., 2021). In parallel, emerging deep-learning RSEI variants demonstrate performance gains but at a cost to interpretability (Gong et al., 2025); our entropy + window approach prioritizes transparency and comparability for policy evaluation. This improved RSEI index for environmental assessment of two 100RC cities (Pune and Surat) and two non-100RC cities (Chattogram and Nagpur), which expands the ecological variables of the ecological index based on remote sensing data from Landsat 8. It used the entropy-based weighted and moving window method, which significantly enhances the spatial and temporal adaptability to overcome the limitation of the traditional RSEI model. The moving window size 133 pixels × 133 pixels followed Wang et al. (2023), which balances local sensitivity and computational efficiency. The innovation of this study lies in combining six ecological indicators: CVI, Wetness, NDBSI, ISI, UI, and LST, with an entropy-based moving window approach that dramatically weights indicators across space. Rather than using only NDVI, NDBSI, Wetness, and LST, this study employed a CVI, which is an interaction among NDVI, EVI, and SAVI. A combination of SAVI and NDVI improved the sensitivity in semi-arid or bare soil (A. Huete et al., 2002).

On the other hand, interaction between EVI helps to correct the NDVI’s weakness in dense forest or humid tropical areas, as EVI is more responsive to canopy structure and less prone to saturation than NDVI. So, CVI enhances discrimination of vegetation health, type, or stress. Healthy and denser vegetation areas are more ecologically healthy than sparse, depleted vegetation. This study also incorporated the ISI and UI, alongside the NDBSI, in calculating the RSEI. When applied to urbanized landscapes, traditional RSEI models, especially those relying solely on NDBSI to represent dryness and anthropogenic disturbance, may suffer from limited accuracy due to spectral mixing between bare soils and built-up areas.

To address this limitation, the ISI and the UI have been proposed as complementary indicators. ISI is specifically designed to capture impervious urban features such as roads and rooftops that are not adequately separated by NDBSI, especially in arid or heterogeneous urban settings (H. Xu, 2008). The inclusion of ISI allows for more granular differentiation of artificial surfaces, thus improving the accuracy of urban ecological assessments. In parallel, the UI integrates spectral responses from multiple bands (e.g., SWIR, NIR) and emphasizes urban morphological structure, thermal characteristics, and land-use intensity, capturing aspects of urban compactness and expansion that NDBSI and ISI alone may miss (Weng, 2009). When UI is incorporated into the RSEI framework, it provides a comprehensive quantification of urbanization-related stress, such as heat absorption and surface sealing, both of which are critical to urban ecological quality (T. Zhang et al., 2021).

Therefore, incorporating ISI and UI into the dryness component of RSEI not only improves urban feature extraction but also strengthens the ecological interpretability of the index, making it a more robust tool for urban sustainability assessment, land-use monitoring, and policy formulation in rapidly urbanizing areas.

Here, Eq. 6 was used to normalize CVI and wetness indicators. NDBSI, ISI, UI, and LST indicators were normalized using Eq. 7. After that, the normalized images were inserted into Eq. 4 for entropy calculation and then into Eq. 5 for weight estimation. The final RSEI estimation equation for this study is as follows:

9 $$RSEI= {W}_{1(i,j)}\times CVI+{W}_{2(i,j)}\times Wetness+{W}_{3(i,j)}\times NDBSI+{W}_{4(i,j)}\times ISI+{W}_{5(i,j)}\times UI+{W}_{6(i,j)}\times LST$$

### Analytical methods

To evaluate the ecological trends and the impact of urban resilience frameworks, several statistical and spatial analytical methods were applied.

#### Two-way ANOVA

A two-way analysis of variance (ANOVA) was performed to examine the effects of city (100RC vs non-100RC), year (2016–2024), and their interaction on RSEI values. This analysis helps determine if resilience strategies significantly influence ecological outcomes over time.

#### Moran’s *I*

Moran’s *I* statistic was calculated to assess spatial autocorrelation and clustering of ecological quality. A high Moran’s *I* value indicates strong clustering of ecological degradation or improvement.

#### Hotspot analysis 

The Getis-Ord Gi statistics* were used to identify clusters of high and low ecological quality (hotspots and cold spots), providing insights into the spatial dynamics of urban ecological degradation.

##  Results

In this study, we evaluate the changes in the ecological conditions of two 100RC cities and two non-100RC cities. We compare Surat (100RC) and Chattogram (non-100RC) cities, as well as Pune (100RC) and Nagpur (non-100RC) cities, since these cities share similar climatic conditions, enabling us to compare them directly. The mean value of RSEI of four 100RC and non-100RC cities over 5 years is shown in Table 3.

**Table 3:** Mean RSEI values of study areas over 5 years

City	Year				
	2016	2018	2020	2022	2024
Chattogram	0.5472	0.4979	0.5117	0.511	0.4851
Surat	0.4726	0.4444	0.4233	0.4093	0.4091
Nagpur	0.4739	0.3663	0.4379	0.4744	0.3416
Pune	0.3869	0.4211	0.4175	0.4505	0.4221

In Chattogram, the ecological condition (as indicated by the RSEI value) has undergone a sudden change. The mean RSEI value decreased sharply from 2016 to 2018 and then increased again in 2020 due to the COVID-19 pandemic. In 2022, the values remained unchanged and then dropped again in 2024. On the other hand, Surat’s ecological condition has undergone a gradual change. The mean value of RSEI of Surat decreased gradually over time.

A similar situation can also be observed between Nagpur and Surat. The non-100RC city of Nagpur experienced a sharp decline in its ecological condition from 2016 to 2018, followed by an improvement during 2020 and 2022, and then a sharp decline again in 2024. The environmental condition in Pune gradually improved between 2016 and 2018, with a slight decrease in 2020. Between 2020 and 2022, the mean RSEI value again increased in Pune and then slightly decreased in 2024.

In summary, the ecological conditions of non-100RC cities are changing abnormally, whereas those of 100RC cities are undergoing a gradual change. This suggests that environmental degradation in 100RC cities is slower than in non-100RC cities, likely due to the implementation of resilient policies.

### 100RC vs Non-100RC ecological condition

In this paper, we have analyzed the ecological conditions of four cities: two 100RC cities and two non-100RC cities. To compare apples with apples, we have chosen similar kinds of cities. For example, both Chattogram and Surat are coastal regions, and a river runs through both cities. For comparison, we compared Chattogram with Surat and Nagpur with Pune, as geographical and climatic conditions significantly impact the ecological environment. For comparison, we performed a pixel-based ANOVA test, spatial autocorrelation analysis, and hotspot analysis to determine the spatial relationship.

The temporal trends in ecological quality between 100RC and non-100RC cities show significant divergence. While non-100RC cities (e.g., Chattogram and Nagpur) exhibited sharp declines and partial recoveries in ecological quality, 100RC cities (e.g., Pune and Surat) displayed more gradual but persistent declines, particularly after 2020. These differences are illustrated by the two-way ANOVA. The results revealed significant main effects of Group (100RC vs non-100RC) and Year, as well as a significant Group × Year interaction for both city pairs (Surat–Chattogram and Pune–Nagpur) (all *p* < 0.001). This indicates that not only do 100RC and non-100RC cities differ in mean RSEI, but these differences vary across years.

### Surat vs Chattogram

**Fig. 3:**
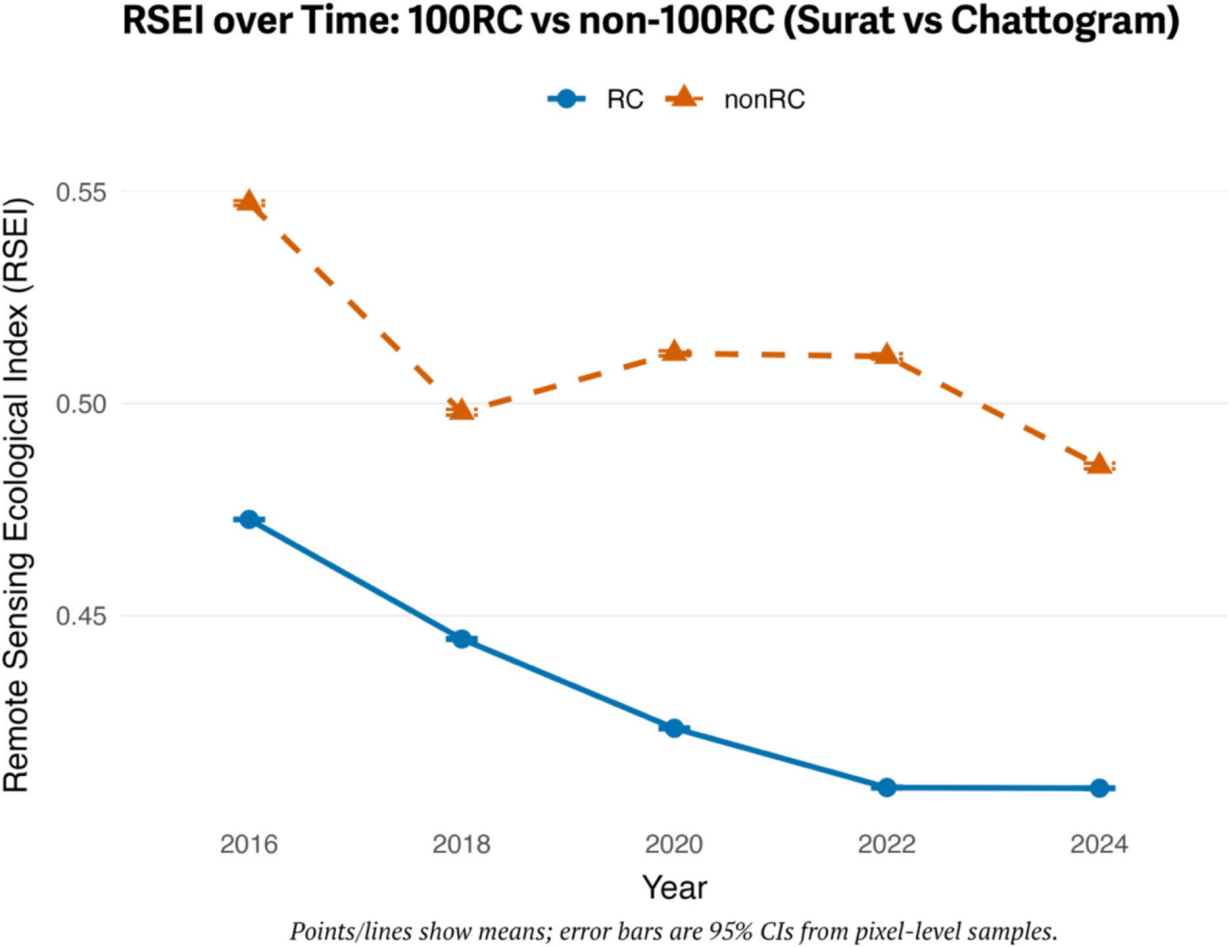
Surat vs Chattogram interaction plot: RSEI over years by group

**Fig. 4:**
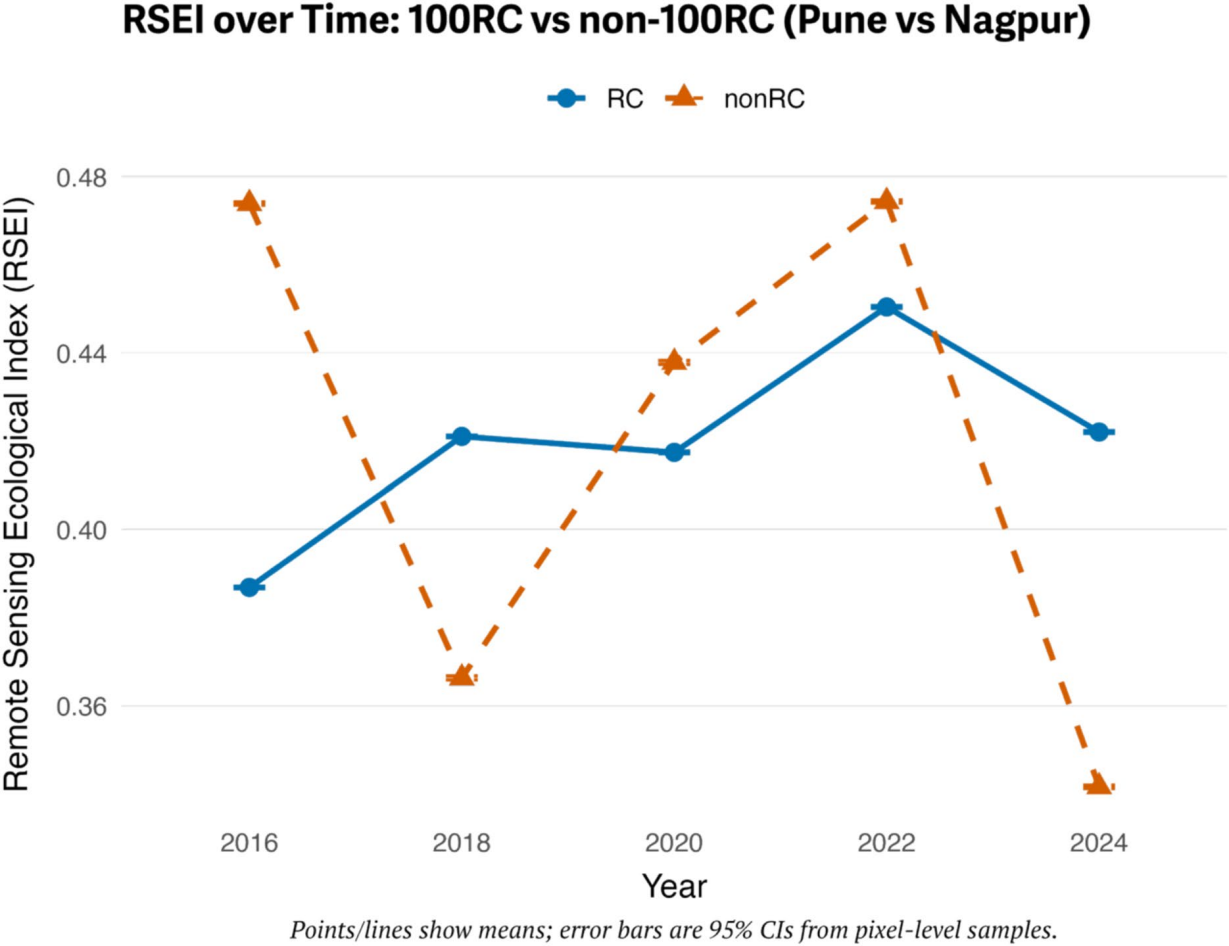
Pune vs Nagpur interaction plot: RSEI over years by group

**Fig. 5:**
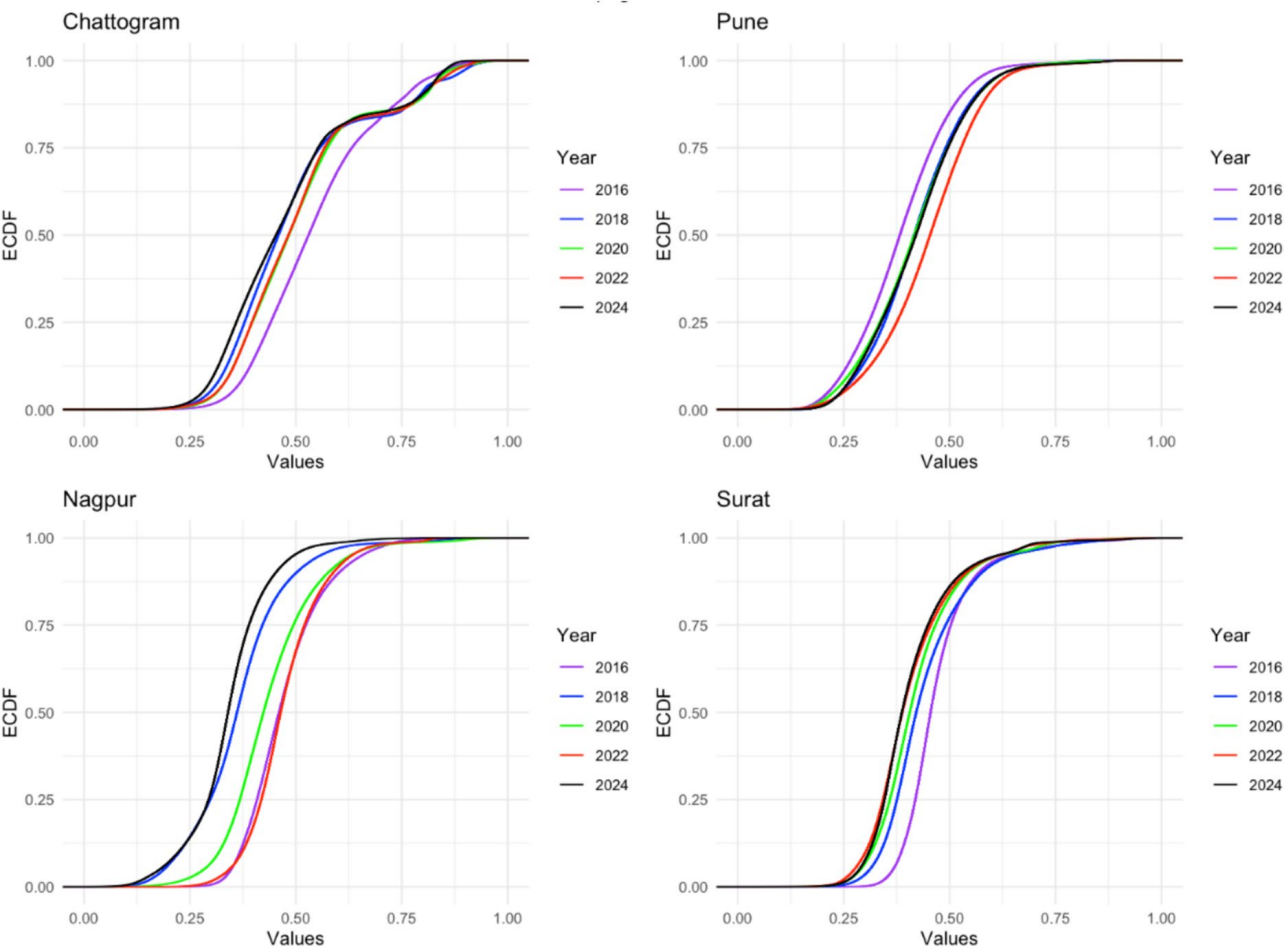
The ECDF of RSEI each year for 100RC and non-100RC cities

Table 4 shows the two-way ANOVA results for Chattogram and Surat. Post hoc comparisons indicate that Chattogram consistently had higher RSEI scores than Surat across all years (*p* < 0.001). Within-group analyses show that Chattogram experienced a fluctuating pattern, with a significant decline from 2016 to 2018, a partial recovery between 2020 and 2022 (which was not statistically significant), and a sharp drop in 2024. In contrast, Surat exhibited an incremental change, with RSEI scores falling consistently until 2022 and leveling off afterward. The interaction plot emphasizes the divergence, with Chattogram showing volatility while Surat displays a gradual deterioration trend (see Fig. 3). Additionally, violin and empirical cumulative distribution function (ECDF) plots illustrate the distributional evidence (Figs. 4 and 5). The violin plots show Chattogram’s distribution widening and shifting across years, indicating instability, while Surat’s distribution steadily compresses at lower values. The ECDF plots confirm these trends: Chattogram’s curves shift right in 2016, left in 2018, slightly right between 2020 and 2022, and then left again in 2024. Surat’s curves shift left consistently until 2022, where they stabilize.

**Table 4:** RSEI for Chattogram and Surat by year, with group comparison results

Year	Chattogram (Non-100RC) mean ± SD	Surat (100RC) mean ± SD	Group comparison (*p*)
2016	0.55 ± 0.08	0.49 ± 0.07	< 0.001
2018	0.50 ± 0.07	0.45 ± 0.06	< 0.001
2020	0.52 ± 0.06	0.42 ± 0.06	< 0.001
2022	0.52 ± 0.06	0.41 ± 0.05	< 0.001
2024	0.48 ± 0.07	0.41 ± 0.05	< 0.001

### Pune vs Nagpur

**Fig. 6:**
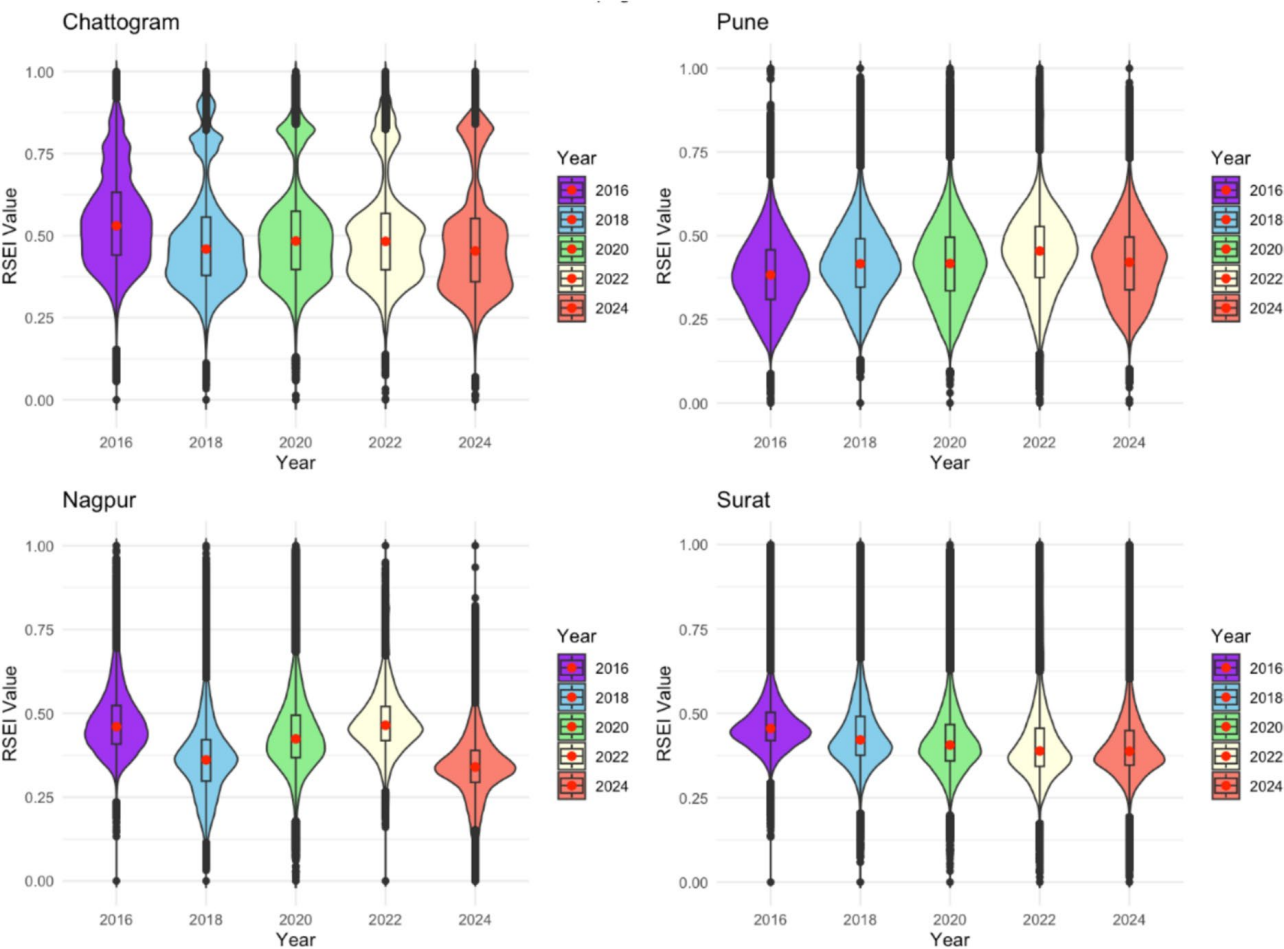
Violin plots showing the distribution of RSEI values in 100RC and non-100RC cities from 2016 to 2024

Similarly, Nagpur showed significantly higher RSEI than Pune in 2016 and 2022 but much lower values in 2018 and 2024 (*p* < 0.001 for all years). Post hoc analyses revealed an unstable ecological pattern in Nagpur, marked by a significant drop in 2018, a temporary recovery in 2022 (which was not significantly different from 2016), and a subsequent collapse in 2024. Pune, on the other hand, exhibited a steadier trend, with an incremental increase up to 2022, followed by a decline in 2024. Table 5 presents the results of the two-way ANOVA, and Fig. 6 shows the interaction plot of Pune and Nagpur. Violin plots illustrate (see Fig. 5) Nagpur’s “boom–bust” cycle, while Pune displays smoother distributional shifts. ECDF curves (see Fig. 4) confirm that Nagpur’s distributions fluctuate dramatically (right in 2016, left in 2018, right again in 2020–2022, left in 2024), whereas Pune’s distributions shift gradually rightward through 2022 before moving leftward in 2024.

**Table 5:** RSEI for Nagpur and Pune by year, with group comparison results

Year	Nagpur (Non-100RC) mean ± SD	Pune (100RC) mean ± SD	Group comparison (*p*)
2016	0.47 ± 0.07	0.39 ± 0.06	< 0.001
2018	0.36 ± 0.06	0.42 ± 0.06	< 0.001
2020	0.44 ± 0.07	0.42 ± 0.05	< 0.001
2022	0.47 ± 0.07	0.45 ± 0.05	< 0.001
2024	0.35 ± 0.06	0.43 ± 0.06	< 0.001

### Comparison summary

The post hoc lettering scheme (Table 6) summarizes these trends. Non-100RC cities (Chattogram, Nagpur) were marked by ecological volatility, characterized by sudden collapses and partial recoveries. 100RC cities (Surat, Pune), in contrast, demonstrated steadier but persistent long-term declines. Taken together, these results suggest that while 100RC cities are less volatile, they are experiencing a structural downward trend in ecological quality, whereas non-100RC cities show unstable ecological trajectories that may undermine long-term environmental resilience. The ECDF and violin plots emphasize that these are not isolated shifts in means but distribution-wide changes, with entire pixel populations moving toward lower RSEI ranges. The significant Group × Year interaction highlights that the 100RC vs. non-100RC gap is time-dependent, varying across years rather than being constant.

**Table 6:** Within-group post hoc summary (letters a, b, c, d, e)

City	2016	2018	2020	2022	2024	Trend summary
Chattogram (non-100RC)	a	b	c	c	d	Fluctuating: drop, partial recovery, fall
Surat (100RC)	a	b	c	d	d	Monotonic decline, plateau at low level
Nagpur (non-100RC)	a	b	c	a	d	Highly volatile: collapse–recovery–collapse
Pune (100RC)	a	b	c	d	e	Gradual rise, then fall

Letters mean years that share the same letter are not significantly different; different letters = significant difference

These patterns suggest that resilience-building in 100RC cities’ resilience strategies may be insufficient to counteract steady degradation, while non-100RC cities face instability that undermines ecological stability.

While temporal trends in RSEI values reveal significant ecological shifts, some of these changes, particularly during the years 2020–2022, may be influenced by external factors. For instance, the COVID-19 lockdowns led to a temporary reduction in industrial activity and vehicle emissions, which likely contributed to brief improvements in air quality and vegetation recovery, particularly in urban areas like Chattogram and Nagpur. Conversely, the post-lockdown rebound in urban activities, coupled with continued land use changes, may explain the sharp decline in 2024. Similarly, changes in land-use policy—such as urban expansion or green-space development initiatives–could have moderated or exacerbated ecological degradation, depending on the timing and effectiveness of their implementation.

### Spatial distribution

**Fig. 7:**
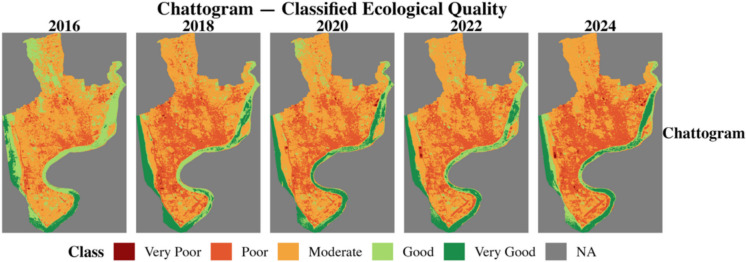
Classified ecological condition of Chattogram (non-100RC)

**Fig. 8:**
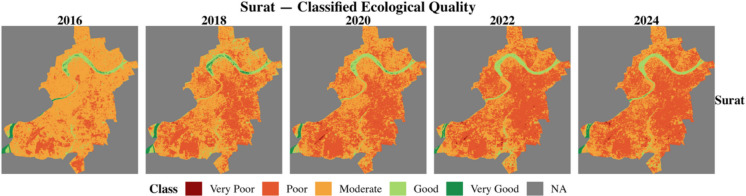
Classified ecological condition of Surat (100RC)

**Fig. 9:**
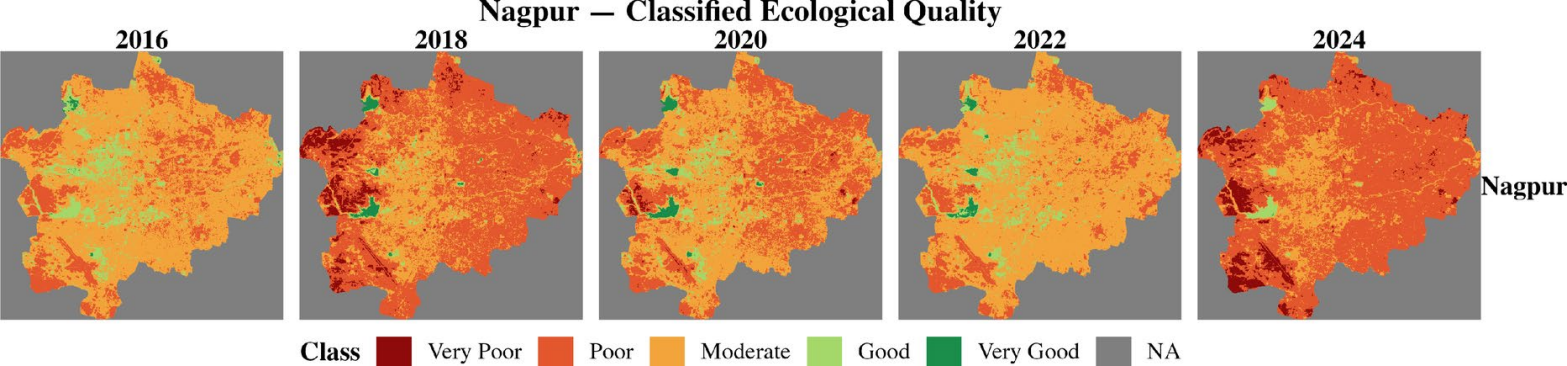
Classified ecological condition of Nagpur (non-100RC)

**Fig. 10:**
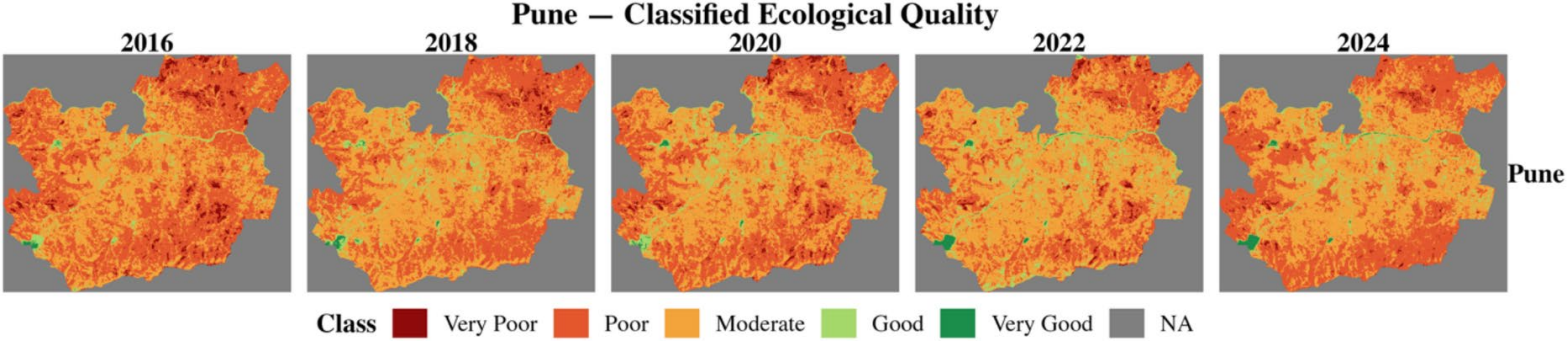
Classified ecological condition of Pune (100RC)

The RSEI of two 100RC and two non-100RC cities was computed using the moving window and weighted entropy method for six environmental indicators. This study numerically examined the ecological conditions from 2016 to 2024 across four cities. This study classified the RSEI values into five categories based on the works of Biswas (2025) and Xu (2013). Table 7 shows the classification of the RSEI value. Figures 7, 8, 9, and 10 show the classified ecological conditions of each city.

**Table 7:** Ecological environment classification

RSEI	Class	Description
0.0–0.2	Very poor	Unfavorable condition for human life to sustain
0.2–0.4	Poor	Poor vegetation and waterbody coverages hinders human life activities
0.4–0.6	Moderate	Fair vegetation and waterbody coverage and somewhat suitable for human life
0.6–0.8	Good	Rich in vegetation and waterbody coverage and suitable for human life
0.8–1.0	Very good	High vegetation-waterbody coverage, rich biodiversity, stable ecosystem

Further, the spatial distribution of RSEI was assessed using Moran’s *I*. The spatial autocorrelation analysis revealed strong positive clustering in all cities, with values ranging from 0.89 to 0.97 (*p* < 0.001), indicating that ecological quality is not randomly distributed but exhibits significant clustering (Table 8). Chattogram (non-100RC) displayed more volatility in ecological clustering, with cold spots expanding dynamically across years. In contrast, Surat (100RC) showed stable clustering of degraded areas, indicating the slower steady decline characteristics of 100RC cities.

**Table 8:** Spatial autocorrelation Moran’s *I* statistics

Year	Surat (100RC)	Chattogram (non-100RC)	Nagpur (non-100RC)	Pune (100RC)
2016	0.943***	0.947***	0.893***	0.920***
2018	0.937***	0.965***	0.924***	0.921***
2020	0.930***	0.957***	0.911***	0.920***
2022	0.938***	0.962***	0.910***	0.916***
2024	0.935***	0.968***	0.920***	0.929***

*p* < 0.001 = ***; *p* < 0.01 = **; *p* < 0.05 = *

**Fig. 11:**
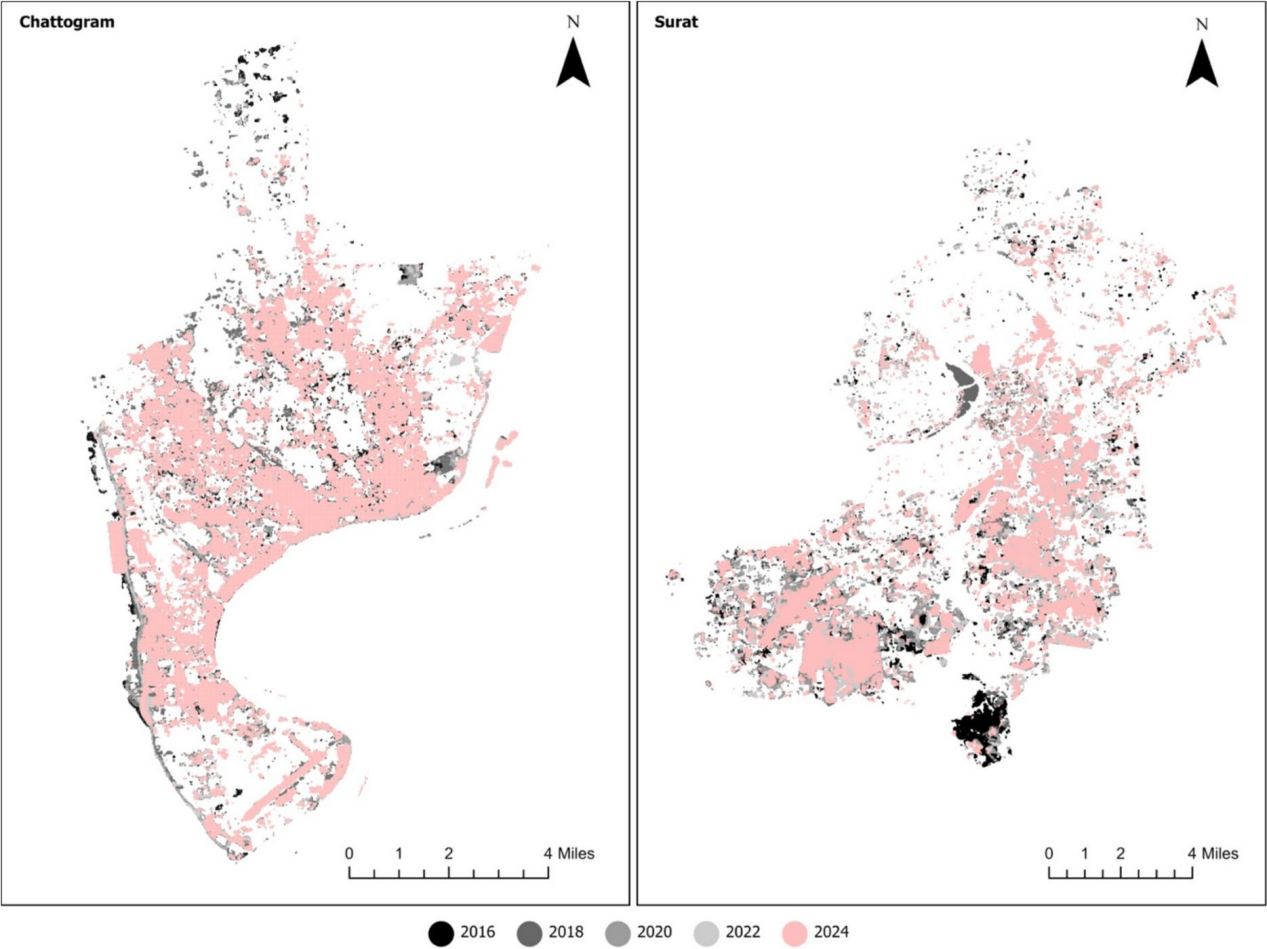
Local spatial dynamics of ecological degradation in Chattogram and Surat

**Fig. 12:**
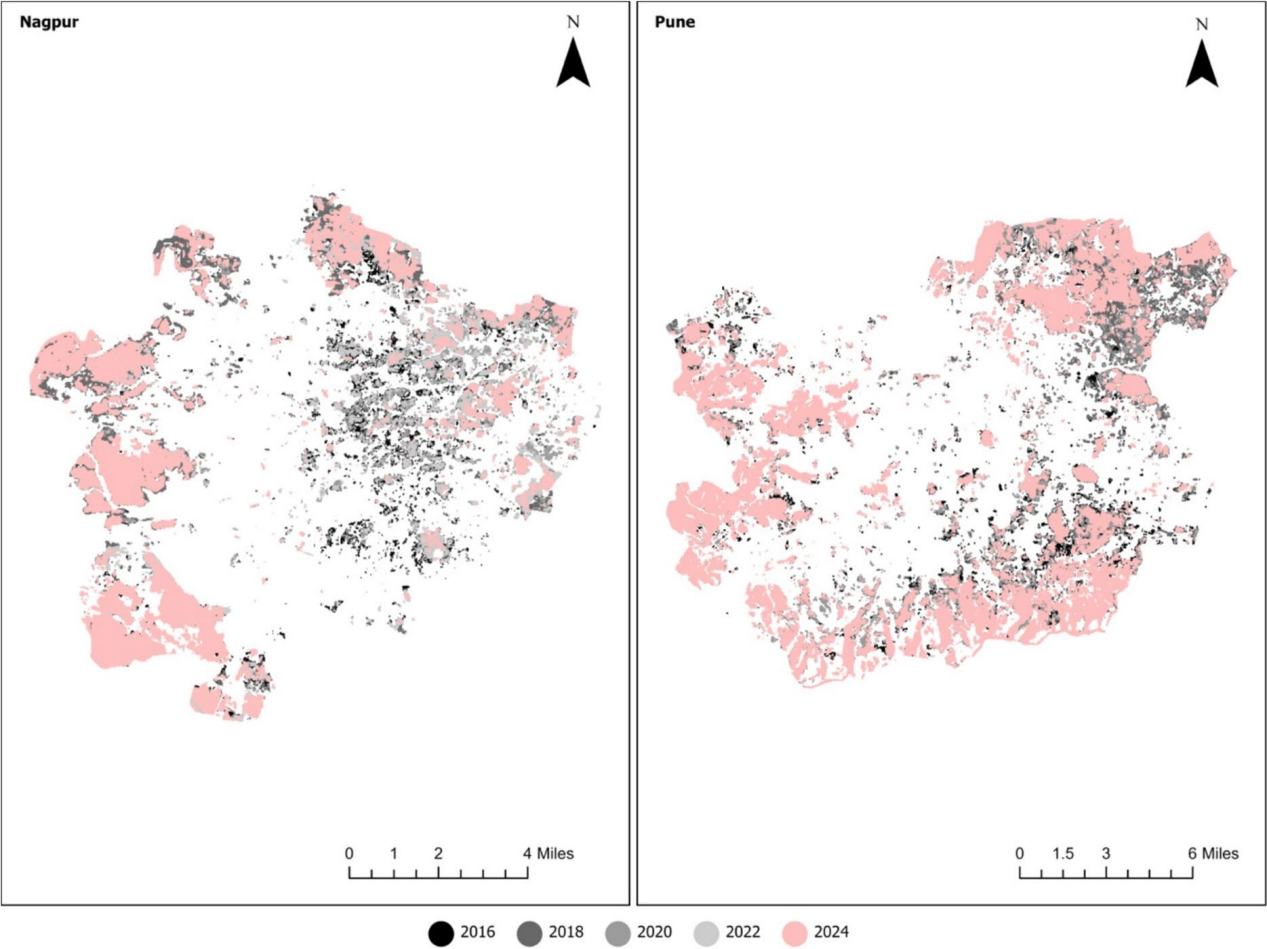
Local spatial dynamics of ecological degradation in Nagpur and Pune

To further understand the local spatial dynamics, a Getis-Ord Gi* hotspot analysis was conducted (Figs. 11–12). This approach identifies clusters of high RSEI values (hotspots) and low RSEI values (cold spots). Figures below show only the cold spots, the ecological degradation concentration, and its spatial distribution over time.

In Chattogram, cold pots were initially limited in extent in 2016 but expanded steadily after 2018, particularly in central and industrial areas. By 2024, large, contiguous cold spot zones had dominated much of the urban core, suggesting intensifying ecological stress and the loss of urban vegetation and water bodies. Surat also displayed persistent cold spots, especially in central and industrial corridors, but here the decline was more gradual and widespread, reflecting a structural downward trend.

Nagpur exhibited a more volatile pattern. Cold spots expanded dramatically in 2018, contracted somewhat in 2020–2022, and then re-emerged strongly in 2024, indicating unstable ecological conditions. Pune, by contrast, showed fewer cold spots before 2020, but their presence expanded after 2022, particularly around the urban periphery. This reflects a gradual yet persistent decline in ecological conditions.

Table 9 summarizes the temporal and spatial analyses. Taken together, Moran’s *I* and hotspot analyses highlight that ecological quality in these cities is both clustered and dynamic. Non-100RC cities (Chattogram and Nagpur) are marked by volatility, with cold spots expanding and contracting sharply across years, reflecting instability in ecological resilience. 100RC cities (Pune and Surat), on the other hand, show steadier trajectories, with cold spots gradually spreading and consolidating over time. The persistence and expansion of cold spots in all cities point to the growing spatial concentration of ecological degradation, reinforcing the evidence from the temporal ANOVA results that urban environmental health is in decline.

**Table 9:** Summary of temporal and spatial analysis

City	Category	Trend (2016–2024)	Volatility	Moran’s *I* (mean)	Key spatial pattern
Chattogram	Non-100RC	Fluctuating decline	High	0.96	Expanding central cold spots
Surat	100RC	Gradual steady decline	Low	0.93	Persistent industrial-core degradation
Nagpur	Non-100RC	Collapse–recovery–collapse	Very High	0.91	Volatile peripheral clusters
Pune	100RC	Rise to 2022 → moderate fall	Moderate	0.92	Cold spots expanding peri-urban fringe

### Resilience strategy analysis

As members of the 100RC initiative pioneered by the Rockefeller Foundation, both Surat and Pune developed comprehensive strategies that extend beyond disaster preparedness to encompass economic, social, and environmental sustainability. These strategies provide valuable insights into how resilience is institutionalized in rapidly urbanizing South Asian contexts. While both cities have adopted a comprehensive framework emphasizing urban environment, mobility, economy, and governance, the quantitative results reveal mixed outcomes in practice.

#### Surat

Surat’s resilience planning^1^ is strongly oriented towards flood resilience, solid waste management, and public health systems, shaped by its history of catastrophic floods and outbreaks. These measures were expected to stabilize ecological quality by reducing pollution and strengthening environmental infrastructure.

However, the RSEI results indicate a steady and monotonic decline in ecological conditions between 2016 and 2022, with stabilization only at lower values in 2024. Hotspot analysis revealed persistent and expanding cold spots across the urban core, while Moran’s *I* values (0.93–0.94) indicated strong clustering of degradation. This suggests that while disaster preparedness (e.g., flood mitigation) may have reduced volatility, long-term environmental pressures from industrialization, waste, and air pollution have outweighed the benefits of resilience interventions. Surat’s strategy appears effective in reducing abrupt ecological shocks, but less effective in halting chronic ecological decline.

#### Pune

Pune’s strategy^2^ is organized into three thematic pillars—growth (integrated planning and housing); environment (climate action, biodiversity protection, and waste management); and economy (skilling, livelihood, and innovation)—encompassing goals, 28 actions, and over 40 projects. Flagship projects include the Mula-Mutha riverfront development, biodiversity parks, and a circular waste economy.

Quantitative results mirror these ambitions but also highlight limitations. Pune’s RSEI trajectory improved consistently from 2016 to 2022, peaking in that year, before declining in 2024. ECDF curves showed steady rightward shifts through 2022, reflecting broad ecological gains, but a leftward shift in 2024 signals a reversal. Hotspot analysis confirms that hotspots persisted longer in Pune than in any other city; however, they weakened after 2022, with new cold spots emerging in peri-urban areas. Moran’s *I* values remained high (0.92–0.93), indicating clustered but stable patterns of ecological quality.

This suggests that Pune’s integration of environmental governance and citizen engagement strategies provided short-term ecological resilience, delaying the expansion of cold spots. However, rapid peri-urban expansion, rising vehicular emissions, and gaps in sewage treatment limited the long-term impact of these measures.

### Resilience and urban sustainability

Both cities’ strategies link resilience building with long-term sustainability goals. Surat emphasizes the importance of resilience in protecting economic vitality and public health from climate risks. Pune’s approach, meanwhile, explicitly includes climate action, biodiversity conservation, and circular economy principles, seeing resilience as a way to achieve a greener and more equitable urban future.

Resilience strategies in Surat and Pune seem to have lessened ecological volatility compared to non-100RC cities. Pune’s integrated environmental governance achieved temporary ecological improvements, while Surat’s focus on flood and public health resilience decreased shock vulnerability but did not stop the ongoing decline. The evidence suggests that 100RC frameworks can help reduce instability, but stronger enforcement, ongoing monitoring, and ecological restoration efforts are necessary to maintain urban environmental quality.

Beyond environmental indicators, institutional and socioeconomic dynamics likely influenced these trajectories. The 100RC cities benefited from enhanced administrative coordination and access to international expertise, improving adaptive planning. In contrast, non-100RC cities face governance fragmentation and limited financial capacity, which increases ecological volatility. Future assessments should explicitly link governance metrics—such as budget allocation and policy implementation rates—with ecological outcomes to better understand urban resilience.

Together, these cases show how 100RC cities in South Asia are developing resilience not only for disaster preparedness but also as a basis for sustainable, inclusive, and adaptable urban growth.

##  Discussion

The improved RSEI developed in this study builds on a decade of progress in ecological environmental assessment using remote sensing. Since Xu (2013) introduced the RSEI framework based on the four core indicators—greenness, wetness, dryness, and heat—numerous studies have refined the model to address its dependency on PCA and its exclusion of water bodies (Biswas, 2025; Liao & Jiang, 2020; Wang et al., 2023; G. Zhang & Kuang, 2025). PCA’s static weighting scheme often limits sensitivity to local heterogeneity and temporal variability, particularly in complex urban environments. The entropy-based weighting used here advances this tradition by capturing spatially adaptive variability while maintaining interpretability. Furthermore, the moving window approach, as suggested by Wang et al. (2023) and Zhu et al. (2020), enhances local responsiveness by simulating the dynamic nature of ecological systems. By extending the RSEI framework to include six indicators – CVI, Wetness, NDBSI, ISI, UI, and LST- this study offers a more comprehensive and responsive tool for analyzing urban sustainability and resilience.

This research contributes to the literature in two important ways. First, it presents an expanded indicator system that captures both vegetation synergy and human-made stress. The CVI, calculated from NDVI, EVI, and SAVI, combines vegetation vitality and canopy density. In contrast, ISI and UI measure impervious surface features and aspects of urban compactness that are not adequately covered in traditional RSEI studies. Second, by comparing 100RC and non-100RC cities in South Asia, this work empirically links urban resilience frameworks to measurable ecological results. To our knowledge, this is one of the first studies in South Asia to combine entropy-weighted RSEI with resilience classification, showing the different effects of resilience planning on ecological stability.

The results corroborate prior findings that resilience frameworks can moderate environmental degradation but often fail to reverse it (Elmqvist et al., 2019; Meerow et al., 2016; Sharifi, 2016). The observed contrast between 100RC and non-100RC trajectories supports the hypothesis that institutionalized resilience strategies reduce ecological volatility while non-100RC cities experience abrupt ecological fluctuations driven by uncoordinated urban expansion. However, the persistence of downward ecological trends in 100RC cities such as Surat and Pune suggests that resilience policies, while stabilizing, remain insufficient to counter structural environmental degradation. These results complement earlier theoretical propositions that resilience is a necessary but not sufficient condition for sustainability, and that it is effective only when ecological restoration and enforcement mechanisms are continuously applied (Elmqvist et al., 2019). Thus, the improved RSEI provides a quantitative bridge between conceptual resilience frameworks and empirical ecological monitoring.

The findings carry several implications for urban sustainability and resilience governance. The comparative evidence indicates that cities in the 100RC network exhibit lower ecological volatility, suggesting that resilience planning stabilizes environmental systems. However, this stability does not equate to ecological recovery—100RC cities show a monotonic but controlled decline, whereas non-100RC cities experience abrupt oscillations. This pattern underscores the need to evolve resilience planning from reactive hazard mitigation toward proactive ecological enhancement. The improved RSEI can serve as a diagnostic tool to evaluate the effectiveness of resilience strategies, enabling planners to identify persistent cold spots and prioritize ecological restoration efforts. By embedding such remote sensing-based indices within a resilience monitoring framework, city governments can establish measurable ecological targets aligned with the Sustainable Development Goals (SDGs 11 and 13).

In comparison with related methodologies such as the fuzzy-AHP RSEI (Alqadhi et al., 2024) and the deep-learning-based RSEI (Gong et al., 2025), the entropy-weighted model proposed here achieves a balance between computational efficiency and interpretability. Its advantage lies in preserving physical meaning while enhancing sensitivity to local changes. Nonetheless, several limitations must be acknowledged. First, the use of Landsat-8 imagery restricts spatial resolution to 30 m, potentially missing fine-scale urban microclimates and small vegetation patches. Second, the temporal resolution (2-year intervals) constrains the detection of short-term fluctuations. Third, the analysis focuses exclusively on biophysical variables, excluding socioeconomic or policy metrics that may strongly influence ecological outcomes. Future research could integrate Sentinel-2 or PlanetScope data, higher-frequency imagery, and ancillary socioeconomic datasets to capture the coupled human–environment dynamics underlying resilience.

Future research should explore how combining improved RSEI outputs with urban form, air quality, or socio-economic indicators can offer a multidimensional perspective on resilience. Longitudinal studies extending beyond 2024 are also needed to distinguish between cyclical ecological fluctuations and structural degradation. Finally, comparative applications in other regional contexts, such as African or Latin American cities, could validate the model’s generalizability and its relevance for global urban sustainability assessments.

##  Conclusion

This study developed and applied an improved RSEI that integrates entropy-based weighting, a moving-window algorithm, and six ecological indicators to assess and compare ecological sustainability in 100RC and non-100RC cities in South Asia. The results reveal that while 100RC cities (Pune and Surat) demonstrate greater ecological stability than non-100RC cities (Chattogram and Nagpur). All cities show a gradual decline in ecological quality between 2016 and 2024. These patterns highlight that resilience frameworks can mitigate volatility but not necessarily reverse long-term environmental degradation.

The research advances current understanding by linking resilience policy frameworks with quantitative ecological metrics, offering a replicable model for monitoring urban sustainability. Methodologically, the inclusion of vegetation synergy (CVI) and urban morphological indices (ISI, UI) enhances the ecological interpretability of RSEI, and the entropy-based moving-window techniques improve spatial precision over traditional PCA approaches. Conceptually, the study provides empirical evidence that resilience and sustainability are interconnected but not synonymous—stability without ecological improvement is sufficient for long-term urban health.

Moving forward, integrating the improved RSEI with socioeconomic, climatic, and governance indicators could enable more comprehensive resilience evaluations. Applying the model to additional time steps and finer-resolution imagery will help capture micro-scale urban dynamics. Ultimately, this research contributes a robust, adaptable framework that urban planners, policymakers, and environmental scientists can use to quantitatively track ecological resilience and guide evidence-based strategies for sustainable urban futures.

## Data Availability

https://github.com/biswasjayanta/100RCImproved-RSEI.git

## Footnotes

1 https://resilientcitiesnetwork.org/surat/

2 https://resilientcitiesnetwork.org/pune/
